# Structure of γ‐secretase‐Ab46 complex: insights into amyloid‐β processing and modulation by the APH‐1B isoform

**DOI:** 10.1002/alz70855_105954

**Published:** 2025-12-24

**Authors:** Lucia Chávez‐Gutiérrez, Mohamed Soliman, Ivica Odorčić, Rouslan Efremov

**Affiliations:** ^1^ VIB‐KULeuven, Leuven, Vlaams Brabant, Belgium; ^2^ VIB‐KULeuven, Leuven, Leuven, Belgium; ^3^ Center for Structural Biology‐VIB, Brussels, Brussels, Belgium

## Abstract

**Background:**

Deposition of amyloid‐b (Ab) peptides in the brain is a hallmark of Alzheimer's disease. Abs are generated through sequential proteolysis of the amyloid precursor protein (APP) by g‐secretase complexes (GSECs). Ab peptide length, modulated by the Presenilin (PSEN) and APH‐1 subunits of GSEC, is critical for Alzheimer's pathogenesis. Despite high relevance, mechanistic understanding of the proteolysis of Ab and its modulation by APH‐1 remain incomplete.

**Methods:**

Here, we report cryo‐EM structures of human GSEC (PSEN1/APH‐1B) reconstituted into lipid nanodiscs in apo form and in complex with the intermediate Ab46 substrate.

**Results:**

We i) found that a divergent loop in APH‐1 is involved together with PSEN1 in substrate‐binding‐induced concerted rearrangements in the enzyme‐substrate complex; ii) characterised the structure of the intermediate Ab46 substrate and its interactions with PSEN1, and iii) showed that polar interactions, including a previously uncharacterised interaction with the PSEN1 loop1, stabilize Ab during GSEC‐ mediated proteolysis.

**Conclusion:**

These findings advance our understanding of the proteolytic mechanisms of GSEC, which is important for the further development of GSEC inhibitors and modulators in cancer and Alzheimer's disease therapies.

